# Primary care physicians’ perspectives on adults with diabetes and the recommended hepatitis B vaccine: A qualitative study

**DOI:** 10.1371/journal.pone.0312168

**Published:** 2024-10-18

**Authors:** Douwné L. Müller, Heather Stuckey, Eileen S. Flores, Li Wang, Thomas Godfrey, William A. Calo, Jessica Yingst

**Affiliations:** 1 Department of Family and Community Medicine, Pennsylvania State University College of Medicine, Hershey, Pennsylvania, United States of America; 2 Department of Public Health Sciences, Pennsylvania State University College of Medicine, Hershey, Pennsylvania, United States of America; 3 Department of Medicine, Humanities, Pennsylvania State University College of Medicine, Hershey, Pennsylvania, United States of America; 4 Penn State Cancer Institute, Hershey, Pennsylvania, United States of America; Onom Foundation, MONGOLIA

## Abstract

**Background:**

People with diabetes are at an increased risk of contracting the hepatitis B virus (HBV). However, hepatitis B (HepB) vaccination rates among adults with diabetes are low. Factors influencing HepB vaccination have not been adequately explored.

**Aims:**

The study aims to identify and understand the barriers adults with diabetes have in receiving the recommended HepB vaccine from the physicians’ perspective.

**Methods:**

This study used semi-structured interviews to ascertain the perspective of 11 primary care physicians (PCPs) in a large academic medical group about HepB vaccination among their patients with Type 1 and 2 diabetes. Thematic analysis yielded descriptions of barriers and strategies that could potentially impact HepB vaccination among adults with diabetes.

**Results:**

Physician responses related to four themes: (1) Conflicting perceptions about HBV risk and the CDC recommendation for adults with diabetes; (2) PCPs don’t perceive HepB vaccination as important as other adult vaccines and prioritize vaccination based on risk exposure; (3) PCPs’ perceived barriers to HepB vaccination among adults with diabetes; and (4) Physician recommended strategies to increase HepB vaccination among adults with diabetes.

**Conclusion:**

Our findings indicate that physicians are generally aware of the existence of the CDC guidelines, but not all physicians recommend the HepB vaccine to adults with diabetes. This is because of a wide variation in treatment concerning glucose monitoring or insulin injection due to varying opinions about actual risk. We also identified barriers adults with diabetes have in receiving the HepB vaccine and strategies to increase HepB vaccination.

## Introduction

Hepatitis B virus (HBV) causes acute and chronic inflammation of the liver. Long-term liver inflammation leads to scarring and loss of liver function. If left untreated, infection causes liver cancer and liver failure [1]. It is often referred to as the "silent epidemic" because many are unaware they are infected until they are screened or damage has occurred [2]. Chronic liver inflammation can lead to serious morbidity, with two out of five people progressing to cirrhosis, liver failure, or liver cancer, and death in one in four cases [3].

People with diabetes (Type 1 and 2) are at an increased risk of contracting HBV. This increased risk for people with diabetes was recognized due to multiple outbreaks among adults with diabetes in long-term care facilities, hospitals, community health centers, ambulatory surgical centers, homes, etc. [4]. Because of these outbreaks, in 2011, the Centers for Disease Control and Prevention (CDC) recommended that all previously unvaccinated adults aged 19–59 years with diabetes be vaccinated against HBV or after a diagnosis of diabetes is made [4,5].

While it is known that vaccination can prevent infection in people with diabetes, current vaccination rates for this population are low. Between 2000–2013, only 20.2% of those with diabetes aged 19–60 years were vaccinated against HBV,[6] while another study reported a vaccination coverage of 24.4% for ages 19–59 in 2015 [7]. Most recently, hepatitis B (HepB) vaccination rates were 33.0% among adults with diabetes aged 19–59 years [8]. Although vaccination rates have increased over the years, it is still not close to optimal. Therefore, it is crucial to understand why adults with diabetes are not receiving the recommended HepB vaccine.

Previous research has been done to identify factors affecting HepB vaccination rates among people with diabetes. Having completed the HepB vaccination schedule decreased with age and increased with income status among adults with diabetes [9]. Vaccination rates varied by ethnicity and factors influencing HepB vaccination included age 40–60 years, lack of education, and foreign birth [6]. Another study found that adults aged 18–59 with diabetes were more likely to be vaccinated against HBV if they were younger, had some college or college education, had been tested for HIV, were a healthcare personnel, or had traveled to hepatitis B virus-endemic areas [10].

Most studies showing factors related to HepB vaccination among adults with diabetes were quantitative; no qualitative research has been done from the physician’s perspective to establish why HepB vaccination rates among adults with diabetes remain low [4–10]. Thus, a qualitative study was chosen to add to the literature on physicians’ knowledge and beliefs of the CDC recommendation, the barriers adults with diabetes experience in receiving the hepatitis vaccine, and strategies to increase HepB vaccination among adults with diabetes from the physician’s perspective. This investigation can provide evidence of gaps and barriers related to HepB vaccination among adults with diabetes and provide a basis for developing effective clinical practices and policies to increase HepB vaccination among adults with diabetes.

## Methods

### Study design and participants

We conducted a descriptive qualitative study to explore the physician’s perspective on HepB vaccination among adults with diabetes. 155 Primary Care Physicians (PCPs) were recruited from the Penn State Health Medical Group in Pennsylvania between February 22, 2022 and April 6, 2022. Purposive sampling was used to recruit physicians by email. The recruitment email asked physicians to complete a screening survey that obtained consent, demographic information, screening questions, and interview scheduling. Three subsequent emails were sent weekly to those who did not respond to the initial invitation. To be eligible for the study, participants had to be physicians (M.D., D.O., & P.A.) from family medicine, family practice, or internal medicine; have a valid Penn State Health email address in the Penn State Health directory; be currently working for Penn State Health; and be fluent in English. No incentives were given to participate in the study. Survey data were collected and managed using REDCap (Research Electronic Data Capture) electronic data capture tools hosted at Penn State Health Milton S. Hershey Medical Center and Penn State College of Medicine [11]. REDCap is a secure, web-based application designed to support data capture for research studies.

### Data collection

Semi-structured interviews (S1 Appendix), approximately 20 minutes in length, were conducted by D.M. over Penn State Zoom. Participants were informed of the purpose of the interview, and verbal consent was obtained. Interviews were recorded via Zoom and transcribed verbatim using professional transcription services (https://www.rev.com//). After transcription, all recordings were deleted. All interviews were conducted by D.M. to establish credibility and homogeneity. D.M. was a female DrPH candidate at the time of the study, with prior interviewing and qualitative analysis experience. There were no prior relationships between D.M. and participants, but participants were aware of the reason for doing the research. No repeat interviews were conducted and no transcripts were returned to participants. D.M. was located in a private office at home and field notes were taken during interviews. Data saturation was obtained as no new themes emerged from the last two interviews.

### Data analysis

This study is presented in line with the Consolidated criteria for reporting qualitative studies (COREQ) checklist (S2 Appendix) [12]. Thematic analysis followed the six stages outlined by Braun and Clarke [13]. Transcripts were read and reread by authors D.M. and E.F. to ensure familiarization. Three transcripts were then independently coded by two team members (D.M. and E.F.) to generate initial codes using an inductive approach. Cohen’s kappa was used to establish interrater reliability at 0.70. Team members met after coding four additional transcripts, and after disagreements were resolved, the rest of the transcripts were coded. Themes were created by looking at codes and combining, clustering, or collapsing them to form categories [13]. This entailed categorizing codes into potential themes based on how different codes could be combined to form overarching themes. Once themes were identified, they were reviewed and refined by D.M. Data management was conducted in NVivo Pro qualitative software (QSR International).

### Trustworthiness of data

To ensure the trustworthiness of this study, the procedures were guided by the four elements of trustworthiness in qualitative research: credibility, transferability, dependability, and confirmability [14]. Trustworthiness was achieved by using purposive sampling, targeting PCPs from a wide range of practices and backgrounds. Team members discussed and met multiple times during data analysis to ensure agreement on codes and themes (investigator triangulation). Thick descriptions were used to describe details on the participants and study methods used to collect data. The two team members who were involved with the data analysis were attentive to reflect on personal assumptions to prevent any subjective or bias interpretations of the findings.

### Ethical considerations

This study was approved by the Pennsylvania State University Institutional Review Board (STUDY00019407). Participants were informed about the aim and procedures of the study and verbal consent was obtained before starting the interview. Confidentiality, privacy, and autonomy were ensured by assigning each participant a code that was used to identify them in the transcripts and manuscript.

## Results

### Participants characteristics

PCPs (n = 11) had a mean age of 50.6 years; 54.5% were male, 81.8% were white, 90.9% were non-Hispanic, and all were from Family Medicine specialty.

### Overall themes of PCPs’ perspective of hepatitis B vaccinations

Participant responses generated four themes; (1) Conflicting perceptions about HBV risk and the CDC recommendation for adults with diabetes; (2) PCPs don’t perceive HepB vaccination as important as other adult vaccines and prioritize vaccination based on risk exposure; (3) PCPs’ perceived barriers to HepB vaccination among adults with diabetes; and (4) Physician recommended strategies to increase HepB vaccination among adults with diabetes. Each theme is outlined in S3 Appendix and briefly described below.

#### Theme 1. Conflicting perceptions about HBV risk and the CDC recommendation for adults with diabetes

Physicians’ opinions differed where risks of contracting HBV were concerned. The majority (63.6%) believed that people with diabetes were at an *“increased risk”* of HBV exposure, referring to sharing of devices or, to a lesser extent, sharing of insulin syringes.

*I know of people within the same family that share the same glucose monitoring devices…-P4*

Other physicians were convinced that not all people with diabetes were at an increased risk and said: “*…it depends*, *I think*, *on how their diabetes being treated…” -P3*

HBV infection was also associated, to a greater extent, with other risk factors such as employment, travel, or risky behaviors.

*…any risks that they would have, either employment wise or personal decision wise, whether they would be increased risk for hepatitis B transmission. -P1*

With regards to the CDC recommendation, physicians that said people with diabetes are at an increased risk for HBV were also the ones that agreed with the recommendation: *“I do agree that they should be given the recommendation*.*”(P10)*, while those that didn’t think there was an increased risk were skeptical of recommending it to everyone with diabetes.

*…most individuals are not necessarily at increased risk for hep B transmission by lifestyle choices. -P1*

#### Theme 2. PCPs don’t perceive hepatitis B vaccination as important as other adult vaccines and prioritize vaccination based on risk exposure

The importance of HepB vaccination among people with diabetes was perceived to be less of a priority compared to other vaccines, with one physician stating:

*…we’re often talking about shingles vaccines or pneumococcal vaccines, flu vaccines, certainly COVID vaccines now. And then hepatitis B falls to the wayside. -P1*

With the HepB vaccine recommendations in mind, physicians tended to recommend the HepB vaccine to patients considered high-risk due to factors other than their diabetes status.

*…we typically would use the hepatitis B vaccine for patients who are higher risk… -P2*

Physicians also mentioned that individuals at risk include “*anybody that’s around healthcare (P2)”* or that *“handles blood or blood products (P6)”*, but did not associate these situations with individuals with diabetes, although they utilize healthcare settings and occasionally handle blood.

Physicians approached the recommendations to people with diabetes to get the HepB vaccine the same way as other vaccines. They mentioned the increased risk of contracting HBV and explained why people with diabetes should receive the HepB vaccine.

*I would just talk about like I do with the other vaccines that are recommended. No, this is recommended for people with diabetes because of their increased risk by getting more needle sticks. -P3*

#### Theme 3. PCPs’ perceived barriers to hepatitis B vaccination among adults within diabetes

*Subtheme 3*.*1*: *Physician-Level Barriers*. **3.1.1. Lack of Physician Knowledge of CDC Recommendation.** Physicians mentioned that some physicians might be unaware of the recommendation for people with diabetes, with one physician saying:

*…part of it is physicians. I think that not everyone is necessarily familiar with the recommendation… -P8*

One physician mentioned a *“lack of knowledge”(P9)*, while another physician suggested that a barrier could be physicians themselves: “*Obviously if a lot of doctors aren’t recommending it…” (P2)*

**3.1.2. Limited Electronic Medical Records (EMRs) Alerts**. Physicians indicated that EMR systems did not prompt them to offer patients with diabetes the HepB vaccine or that a follow-up vaccine was needed.

*They don’t automatically say, “Hey, did this person get their?” But they do it for other things like their eye exams, their foot exams, things like that. It’s not included in that type of recommendation in the recommendations pad, panel. -P8*

Two physicians said that patient vaccinations could be found in a specific section in the EMR system, but it requires physicians to go there and review the information, which could be time-consuming.

*So pretty much the practice is to just look. So, you look for every patient and see what do they due for, and whatever they due for, you recommend. -P9*

A lack of EMR system follow-up reminders contributes to patients and physicians forgetting about follow-up vaccines despite people with diabetes seeing physicians every 3–6 months. One physician said:

*You would think it would be easier given someone with diabetes is coming back fairly regularly… -P10*

Follow-up vaccines were also challenging to complete if not scheduled in advance, leading to physicians and patients forgetting again. One physician mentioned lack of reminders being sent to patients and said:

*…We don’t have the record, or the schedule where we send our reminder to our patients. -P7*

**3.1.3. Competing Priorities.** Time constraints lead to physicians prioritizing information and treatment topics concerning diabetic care and education. This leads to physicians focusing on vital information and the HepB vaccine discussion falling by the wayside.

*more attention towards other things related to their health… other aspects of their care, just their plain diabetes management, their other comorbid health condition management… -P1*

One physician declared it’s not a priority since it’s more considered for those at higher risk.

*…is just we’re not thinking about it as much as a priority. Cause again, traditionally, training for most docs is that it’s more just certain high-risk populations. -P2*

*Subtheme 3*.*2*: *PCPs’ Perception of Patient-Level Barriers*. **3.2.1. Lack of Insurance Coverage.** Physicians mentioned costs associated with getting the vaccine, and one physician stated: “*insurance companies won’t cover it for most people” (P6)*. Some physicians said that patients may “*worry about whether their insurance is going to pay”* (*P4*) for the vaccine or whether a co-payment would be necessary, while others did not know whether insurance would cover HepB vaccines.

*I will admit that I don’t have a good sense of insurance coverage for hepatitis B vaccinations…-P3*

**3.2.2. Patient Beliefs and Vaccine Hesitancy.** Physicians stated that patients exhibit vaccine hesitancy across the board and that it’s most likely due to “*a fear of the needles” (P7)* or side effects, health literacy, and misinformation.

Vaccine hesitancy has also been exacerbated by the COVID-19 vaccine controversies during the global pandemic, with one physician stating: *“there are some people that have become more vaccine hesitant overall because of COVID vaccines” (P3)* and another saying: “*It’s got everybody concerned about the safety*, *what’s in these vaccines*. *A lot more people hesitant to want to get it (P10)*. It has also made it harder for physicians to recommend any vaccines. Vaccine fatigue because of COVID was also mentioned by physicians and can be described as the effect multiple vaccine recommendations have on people’s ability to take action.

*…there’s like a sense of vaccine fatigue right now from patients, because it’s so prominent and present…-P2*

**3.2.3. Lack of Patient Knowledge and Health Literacy.** According to some physicians, patients may not be aware of HBV and why they need to vaccinate against it. Patients may push back or think they don’t have to worry about HBV since they don’t consider themselves at high risk.

*…patient themselves not understanding why it’s necessary…-P6*

One physician also mentioned that there might be *“a knowledge gap” (P4)* in understanding the importance of HepB vaccinations, while another said that *“health literacy is a major challenge” (P7)* and that individuals may not understand their diabetes and therefore do not understand *“what is hepatitis B or why is that important*?*” (P7)*

**3.2.4. Socio-demographics.** Lower education was mentioned as a barrier, with physicians linking lower education to being uninsured or underinsured. These patients experience barriers to accessing care, which may cause them to utilize multiple healthcare resources, leading to inconsistent care. This could explain why individuals are unvaccinated or have incomplete vaccinations.

*…those with less education, they’re more likely to be uninsured or underinsured and, therefore not have access to preventative health and seeing a primary care provider–P1*

Age was also mentioned, with most physicians stating that younger individuals would be more likely vaccinated against HBV due to childhood immunizations and that older individuals may have been left by the wayside.

*I think the younger groups would have gotten the hepatitis B as part of their childhood immunization… Whereas, I think the older population, there would be some that haven’t gotten it when they were children…-P7*

Two physicians stated that older adults are more reluctant to receive vaccines, with one saying: *“patients as they age are probably less likely to take the vaccine” (P9)*, while another believed older adults are more receptive to vaccinations than younger individuals: “*the older population is more receptive to vaccinations in general*, *and it’s definitely more of the younger population that I do have more challenges with” (P2)*

Other demographic characteristics mentioned as barriers to getting the HepB vaccine were religion, gender, race, and socioeconomics, with one physician stating that:

*I would say gender not so much… Race maybe in lower SES status groups, which may tend to be certain races, just in general. But I don’t know if it’s more the race, or it’s more the socioeconomic status piece. My suspicion is it’s more the SES piece, and not the race directly. -P2*

#### Theme 4. Physician recommended strategies to increase hepatitis B vaccination among adults with diabetes

*4*.*1*. *Physicians’ Strategies*. Some physicians suggested *“educating primary care physicians” (P1)* about the CDC recommendation and why it’s necessary to recommend the HepB vaccine to patients with diabetes. One physician said: *“reinforcing and giving more education at the provider level first*” *(P2)* was needed, while another stated:

*…we need more physician education… I don’t think it’s widely understood as to why this is being recommended. -P6*

Another strategy mentioned by physicians was implementing a *“quality measure”* to encourage physician adherence in recommending the HepB vaccine. One physician said that their office uses quality metrics to help keep track, but that HepB vaccination wasn’t one of them and recommended:

*…getting that added into our quality measures and into our checklist that we do with diabetic patients… -P3*

In addition, physicians mentioned different resources to help them recommend the HepB vaccine. Multiple physicians suggested programming EMR alerts to prompt HepB vaccine recommendations and would serve as reminders for physicians. In addition to EMR alerts, one physician suggested including it in the guidelines:

*I think it would be really good if it is included in the guidelines… -P7*

One physician suggested including it in diabetes education to educate patients at the practice, while another two physicians suggested including medical assistants in reviewing information and processing the HepB vaccine before the physician sees the patient.

*…having it within a diabetes education visit may be a more beneficial process where the nurse or the dietician doing the diabetes education have that as part of their education… -P1*

*if we had a care protocol that allowed our nurses to propose the order because there was a care gap identified*, *the physicians would be more willing to sign that … -P4*

*4*.*2*. *Patient Strategies*. Physicians also mentioned multiple strategies to improve HepB vaccination among people with diabetes. One physician suggested using information resources like brochures, flyers, and videos for patients at the practice, while others suggested using advertisements and creating awareness on social media.

*… a flyer or a brochure. If we could develop some kind of a video or something, they can watch, they can explain… -P7*

*If you put it on T*.*V*., *on commercials and say*, *"This is something you should do*, *ask your doctor*,*" -P6*

One suggestion was to get insurance companies to incentivize their clients to get the HepB vaccine. Reducing insurance costs for clients who use vaccines or other preventative medicine may persuade people to receive recommended vaccines.

*What will work is economic incentive… An insurance company that says, oh yeah, we’re going to drop your rates if you do this…-P9*

Another strategy was to have pharmacies promote the vaccine more, while one physician suggested getting community organizations involved, organizing community fairs to educate individuals, or doing vaccination clinics.

*…organizing community camps regarding, let’s say preventative thing, vaccinations for pneumonia, for hepatitis B…-P5*

## Discussion

This study sought to understand physicians’ knowledge and beliefs about the CDC recommendation, to investigate the barriers adults with diabetes experience in receiving the HepB vaccine, and strategies to increase HepB vaccination from physicians’ perspectives. Existing literature on identifying barriers is limited, and our study adds to the literature regarding HepB vaccination barriers among adults with diabetes. To the best of our knowledge, this is the first qualitative study to investigate HepB vaccination among people with diabetes, the barriers associated with HepB vaccination, and the strategies to overcome the barriers from a PCP’s perspective.

The physicians interviewed were all aware of the existence of the CDC recommendation that previously unvaccinated adults aged 19–59 years with diabetes be vaccinated against HBV [4,5]. However, not all physicians agreed that adults with diabetes are at an increased risk of contracting HBV and, therefore, were skeptical about recommending the HepB vaccine to all adults with diabetes or recommending it at all. The skepticism was due to physicians considering how patients treat their diabetes. Patients treating their diabetes with diet and exercise, oral medication, or continuous glucose monitoring weren’t considered high risk since their glucose monitoring, or insulin injection exposure was less. Physicians also mainly focused on recommending the HepB vaccine to individuals considered high-risk, such as healthcare workers and people traveling, rather than their diabetes status. In adults with diabetes, more critical vaccines such as the flu, pneumonia, shingles, and now Covid were considered more important than the HepB vaccine.

The physicians who participated in the study also listed several barriers that could explain the low vaccination rates and why adults with diabetes are not receiving the recommended vaccine. Barriers that affected physicians included a lack of physician knowledge of the CDC recommendation, limited EMR alerts, and competing priorities. Patient barriers included lack of insurance coverage, patient beliefs and vaccine hesitancy, lack of patient knowledge and health literacy, and socio-demographics. These results were similar to previous research on barriers to adult vaccination. According to Johnson et al., [15] the most common reasons for consumers not vaccinating were a lack of physician recommendations and incorrect assumptions such as healthy people do not require vaccination [15]. Healthcare providers’ perceived barriers to vaccination included patients’ lack of regular check-ups, a lack of a reminder system, patients’ dislike of needles, fear of side effects, lack of insurance coverage, and lack of knowledge about disease prevention [15]. Another study found a variety of reasons for low adult vaccination rates, including undervaluation of adult vaccination, a lack of public and provider knowledge about adult vaccination, a lack of insurance coverage, a lack of knowledge about the safety and efficacy of adult vaccines, patterns of health care use by adults, insufficient infrastructure to support adult vaccination, and complex public health recommendations [16]. Future research is required to explore adults with diabetes’ awareness and knowledge of the recommended HepB vaccine and the barriers they have to receiving the recommended HepB vaccine from the individual’s perspective.

When looking at socio-demographics as a barrier specifically, our study found that physicians thought younger individuals would be more likely to be vaccinated against HBV due to childhood immunizations and that older individuals may have been left by the wayside. This is similar to previous research that found that younger adults with diabetes were more likely to be vaccinated against HBV than older adults [6,9,10]. Also, previous research has shown that individuals with higher education had higher HepB vaccination rates than those with less education [6,10,17]. We found that physicians felt that individuals with less education are more likely to be uninsured or underinsured and may experience barriers to accessing care. Also, lower health literacy may play a role and make it challenging to manage their secondary health issues, such as vaccine status, in addition to their primary diabetes diagnosis. In a study by Villarroel and Vahratian [9], they discovered that completion of the HepB vaccination series increased with income status. Our study indicated that socioeconomic status plays an essential part in the continuity of care and availability of resources. Diabetes care involves team-based care, and it’s difficult for the uninsured or underinsured to access care, which could explain low vaccination rates. Research is needed to investigate effective ways to increase HepB vaccination among individuals with diabetes.

Lastly, physicians provided strategies to increase HepB vaccinations among adults with diabetes. Physicians described that educating physicians on the CDC recommendation and why it is necessary for adults with diabetes to get the HepB vaccine would be beneficial. Implementing quality measures to encourage physicians to adhere to the HepB vaccine recommendation and making it part of the guidelines for physicians could increase vaccination. Physician resources to help physicians recommend the HepB vaccine included programming EMR alerts to prompt HepB vaccine recommendations that would serve as reminders for physicians. Another strategy was to use diabetes educators or informing individuals within a diabetes education session. Nurses would educate and inform adults with diabetes about the recommendation and even schedule the HepB vaccinations. Also, allowing medical assistants to review the information and process the HepB vaccine before the physician sees the patient would be beneficial. The last strategies mentioned were patient resources. This included providing materials for patients at the practice, using advertisements and creating awareness on social media, getting insurance companies and pharmacies involved, organizing community events to inform individuals, and even having vaccine clinics at events.

The results of this study highlight the importance of understanding and addressing the barriers adults with diabetes have in receiving the HepB vaccine. Recommendations prompted by this study include increasing awareness of the CDC recommendation among healthcare professionals and people with diabetes, developing practice procedures to help physicians recommend the vaccine, and making the recommendation part of the standard of care for adults with diabetes. To effectively enhance HepB vaccination among adults with diabetes, physicians must recommend the CDC recommendation to everyone with diabetes.

Limitations related to this study include the sample’s representativeness, which only consisted of primary care physicians from family medicine, and other healthcare providers were not included. The findings also reflect only physicians’ perspectives on HepB vaccination and barriers to HepB vaccination among adults with diabetes and do not include the views of people with diabetes. The small sample size, a consequence of a qualitative study, could threaten the validity and generalizability of the results. Since the researcher was involved in the interviews, social desirability bias may have occurred. Lastly, during the COVID-19 pandemic, there was limited time to interview physicians, and it may also have influenced vaccination procedures in the healthcare system. Despite these limitations, the findings provide a unique look at how physicians understand and implement the CDC’s recommendation for the HepB vaccine for adults with diabetes.

## Conclusion

This study improves our understanding of physicians’ knowledge and beliefs about the CDC recommendation, the barriers adults with diabetes experience in receiving the HepB vaccine, and strategies to increase HepB vaccination. The findings indicate that providers are aware of the CDC recommendation but that some physicians may be sceptical in recommending it because of how some treat their diabetes. We also identified the barriers adults with diabetes have in receiving the HepB vaccine, similar to previous research. Physicians also provided strategies to increase HepB vaccination among adults with diabetes. This research supports the need for implementing organizational and structural changes within the practice to help physicians effectively promote HepB vaccination among adults with diabetes as well as increasing awareness of the CDC recommendation among healthcare professionals and people with diabetes.

## Supporting information

S1 Appendix Interview guide. (DOCX)

S2 Appendix Consolidated criteria for reporting qualitative studies (COREQ): 32-item checklist. (DOCX)

S3 Appendix Outline of themes, subthemes, and examples of quotes. (DOCX)
