# Supplementary material for: Primary care physicians’ perspectives on adults with diabetes and the recommended hepatitis B vaccine: A qualitative study
Source: PLoS One. 2024 Oct 18;19(10):e0312168. doi: 10.1371/journal.pone.0312168 (PMC11488695; doi:10.1371/journal.pone.0312168)
Supplement: S1 Appendix — (DOCX) [file pone.0312168.s001.docx]

**S1 Appendix**

**Interview Guide**

Good Afternoon Dr. xxx,

Thank you for taking the time to participate in this interview today. The interview will take approximately 20 minutes of your time and I’ll be asking questions about hepatitis B vaccination barriers among adult patients with diabetes. Just to remind you this interview will be recorded and I’ll inform you as soon as I start the recording.

Please do not provide any identifying information about other individuals when providing answers.

I’ve sent you the summary explanation of research with the recruitment email, have you had the chance to review it?

*If no, review with participant and inform them that you will start recording.*

*If yes, inform them that you will start recording.*

**START RECORDING**

State date, time of interview. State participant interview number.

Can you confirm that we’ve discussed the summary explanation of research for this study, and you give permission for audio and video recording?

**OPENING QUESTIONS**

1. What are your thoughts on hepatitis B infections among adults with diabetes? Specifically, do you think they are at increased risk of getting hepatitis B?
   1. Are your thoughts different for type 1 or type 2 diabetes?
2. Do you believe adults with diabetes should receive the recommended hepatitis B vaccine? Why or why not?

**HEPATITIS B VACCINE BARRIERS**

1. What barriers prevent those with diabetes in getting the hepatitis B vaccine?
2. Why do you think it is difficult for patients with diabetes in receiving the follow up doses for the hepatitis B vaccines?
3. What are your thoughts on age and hepatitis B vaccination rates among those with diabetes? Specifically, do you see a difference between younger and older individuals?
   1. What will be your recommendation for younger adults with diabetes? And for older adults with diabetes?
4. Also, what are your thoughts on why adults with diabetes with less education are less likely to be vaccinated against hepatitis B?
   1. What is your approach in recommending the hepatitis B vaccine to those adults with diabetes that are less educated?
5. Are there any other barriers that you think that inhibit adults with diabetes in getting the hepatitis B vaccine?

- Probe: Your thoughts on gender, race, income, place of birth, insurance

1. What do you say to a patient with diabetes to describe why the Hepatitis B vaccine may be important?
2. What do you tell them if they are reluctant?  Does this seem to help?

Is there anything you would like to add, other thoughts or concerns?

Thank you so much for participating in the study, I will stop the recording now.

**STOP RECORDING**

If you think of any questions, please feel free to reach out.

Thank you and hope you have a great rest of your day.
