# Supplementary material for: Primary care physicians’ perspectives on adults with diabetes and the recommended hepatitis B vaccine: A qualitative study
Source: PLoS One. 2024 Oct 18;19(10):e0312168. doi: 10.1371/journal.pone.0312168 (PMC11488695; doi:10.1371/journal.pone.0312168)
Supplement: S2 Appendix — (DOCX) [file pone.0312168.s002.docx]

**S2 Appendix**

**Consolidated criteria for reporting qualitative studies (COREQ): 32-item checklist**

No Item Guide questions/description Reported on Page #

**Domain 1: Research team and reflexivity**

Personal Characteristics

1. Interviewer/facilitator Which author/s conducted the interview or focus group? Pg 6

2. Credentials What were the researcher’s credentials? *E.g. PhD, MD* Pg 6&7

3. Occupation What was their occupation at the time of the study? Pg 6&7

4. Gender Was the researcher male or female? Pg 6

5. Experience and training What experience or training did the researcher have? Pg 7

Relationship with participants

6. Relationship established Was a relationship established prior to study commencement? Pg 7

7. Participant knowledge of the What did the participants know about the researcher? *e.g. personal goals,*

Interviewer *reasons for doing the research* Pg 7

8. Interviewer characteristics What characteristics were reported about the interviewer/facilitator? e.g. *Bias,*

*assumptions, reasons and interests in the research topic* Pg 7

**Domain 2: study design**

Theoretical framework

9. Methodological orientation and What methodological orientation was stated to underpin the study? *e.g.*

Theory *grounded theory, discourse analysis, ethnography, phenomenology, content analysis* Pg 6

Participant selection

10. Sampling How were participants selected? *e.g. purposive, convenience, consecutive, snowball* Pg 6

11. Method of approach How were participants approached? *e.g. face-to-face, telephone, mail, email* Pg 6

12. Sample size How many participants were in the study? Pg 9

13. Non-participation How many people refused to participate or dropped out? Reasons? N/A

Setting

14. Setting of data collection Where was the data collected? *e.g. home, clinic, workplace* Pg 7

15. Presence of non-participants Was anyone else present besides the participants and researchers? Pg 7

16. Description of sample What are the important characteristics of the sample? *e.g. demographic data, date* Pg 8

Data collection

17. Interview guide Were questions, prompts, guides provided by the authors? Was it pilot tested? Pg 6

18. Repeat interviews Were repeat interviews carried out? If yes, how many? Pg 7

19. Audio/visual recording Did the research use audio or visual recording to collect the data? Pg 6

20. Field notes Were field notes made during and/or after the interview or focus group? Pg 7

21. Duration What was the duration of the interviews or focus group? Pg 6

22. Data saturation Was data saturation discussed? Pg 7

23. Transcripts returned Were transcripts returned to participants for comment and/or correction? Pg 7

**Domain 3: analysis and findings**

Data analysis

24. Number of data coders How many data coders coded the data? Pg 7

25. Description of the coding tree Did authors provide a description of the coding tree? N/A

26. Derivation of themes Were themes identified in advance or derived from the data? Pg 7

27. Software What software, if applicable, was used to manage the data? Pg 7

28. Participant checking Did participants provide feedback on the findings? N/A

Reporting

29. Quotations presented Were participant quotations presented to illustrate the themes / findings? Was each

quotation identified? *e.g. participant number* Pg 8-18

30. Data and findings consistent Was there consistency between the data presented and the findings? Pg 8-18

31. Clarity of major themes Were major themes clearly presented in the findings? Pg 8-18

32. Clarity of minor themes Is there a description of diverse cases or discussion of minor themes? Pg 8-18

Developed from:

Tong A, Sainsbury P, Craig J. Consolidated criteria for reporting qualitative research (COREQ): a 32-item checklist for interviews and focus groups. International Journal for Quality in Health Care. 2007. Volume 19, Number 6: pp. 349 – 357
