# Supplementary material for: Primary care physicians’ perspectives on adults with diabetes and the recommended hepatitis B vaccine: A qualitative study
Source: PLoS One. 2024 Oct 18;19(10):e0312168. doi: 10.1371/journal.pone.0312168 (PMC11488695; doi:10.1371/journal.pone.0312168)
Supplement: S3 Appendix — (DOCX) [file pone.0312168.s003.docx]

**S3 Appendix.**

**Outline of Themes, Subthemes, and Examples of Quotes**

| **THEME & SUBTHEMES** | **CODES** | **N** | **EXAMPLES** |
| --- | --- | --- | --- |
| **Theme 1. Conflicting perceptions about HBV risk and the CDC recommendation for adults with diabetes.** | | | |
|  | 1. Perception of Risk Level | 11 | *“My understanding is yes, that patients who have diabetes are at a higher risk of hepatitis B... My understanding of it is that because of all of the percutaneous finger sticks and all of the equipment required and all the exposures, they have an increased rate of exposure, which results in increased rate of infection over the normal population.”* ***–Participant 11***  *“So certainly, it depends, I think on how their diabetes being treated. So, if they are on medication and they are not regularly checking their blood sugars, I don't think that their risk is really increased at all. But if they are regularly doing finger sticks injections, there is more potential for hepatitis B infection because of the blood-born nature of the disease.”* ***–Participant*** ***1***  *“I'm not sure I would classify them as a higher-risk patient, just for the hepatitis B vaccine.* ***–Participant 2*** |
|  | 1. Perception of CDC recommendations | 11 | *“I do agree that they should be given the recommendation.”* ***–Participant 10***  *“On one hand, I think they should because of CDC recommendations, but on the other hand, like I said, a lot of individuals that have not been vaccinated otherwise because of travel or their occupation, that most individuals are not necessarily increased risk for hep B transmission by lifestyle choices.”* ***–Participant 1***  *“So, if you're in a care institution, you're getting multiple finger sticks and people might not be as careful about cross contamination, I could understand that. Average diabetic out in the community, who's only using their test on themselves is not at risk and besides which we've gotten now to continuous glucose monitoring… So still, I'm not convinced that it needs to be something that we have to make an effort to do.”* ***–Participant 6*** |
| **Theme 2. PCPs don’t perceive hepatitis B vaccination as important as other adult vaccines and prioritize vaccination based on risk exposure** | | | |
|  | - 1. Importance of HBV vaccine for adults with diabetes | 4 | *“Especially with me personally and professionally feel that out of that giant list of adult vaccines that Hep B, is falling at the bottom with the importance for me, for my patients.“* ***–Participant 1*** |
|  | - 1. Other Vaccines | 3 | *“And then when we are talking about vaccines, we're often talking about shingles vaccines or pneumococcal vaccines, flu vaccines, certainly COVID vaccines now. And then hepatitis B falls to the wayside.”* ***–Participant 1*** |
|  | 1. Vaccine Use | 3 | *“I'm a primary care doctor, so we typically would use the hepatitis B vaccine for patients who are higher risk, which naturally includes people in healthcare settings, people that work in prisons, anybody that's around healthcare. A lot of people also that are traveling, it's recommended and required as well, depending upon what area of travel. So that's one of the utilities that we would use that a lot in our adult population, from my perspective.”* ***–Participant 2***  *“I offer hepatitis B vaccine to people who might otherwise be at risk certainly, healthcare personnel and anyone who handles blood or blood products. But I've given people the hepatitis B vaccine for just various, they're traveling overseas or whatever.”* ***–Participant 6*** |
|  | 1. Recommendations for patients | 5 | *“Certainly, if somebody is at risk for hepatitis B through contact, either through their job or their personal choices with blood products or other contractable body fluids from other individuals, that's when I would bring it up. Or if someone is starting to have renal disease and thinking about dialysis or fatty liver disease...”* ***–Participant 1***  *“Usually, this is sort of similar for all vaccines, I make the recommendation. I say, usually this is sort of when I'm going through a preventative health visit or something like that…”* ***–Participant 8*** |
|  | 1. Recommendation for People with diabetes | 9 | *“Usually, I would just talk about like I do with the other vaccines that are recommended. No, this is recommended for people with diabetes because of their increased risk by getting more needle sticks. And I usually keep it pretty simple.”* ***–Participant 3***  *“Yeah. If I was going to recommend a vaccine for somebody that has higher risk for infection of any disease, I would just say from a preventative standpoint, because you are higher risk with diabetes and then if they have other conditions, I would say this is recommended for prevention, because hepatitis B can lead to these outcomes, and then discuss the outcomes of hepatitis, what it can do to your health and how it can impact you. And again, generally focus on the prevention part. We want to prevent illnesses from happening. We want to be ideally not taking care of them after the fact, because that can be even worse for the patient. Bad outcomes, clinically.”* ***–Participant 2*** |
| **Theme 3. PCPs’ perceived barriers to hepatitis B vaccination among adults with diabetes.** | | | |
| ***Subtheme: Physician-Level Barriers*** | | | |
| **3a. Lack of Provider Knowledge of CDC Recommendation.** | 1. Doctors not recommending | 2 | *“Obviously if a lot of doctors aren't recommending it, that's the biggest barrier because we're kind of the preventative touch point. So, the provider themself, if they're not recommending it.”* ***–Participant 2***  *“It's hard to say, because I think at this point, our biggest barriers, just that we don't ask patients regularly if they wanted to have had it, etc.”* ***– Participant 3*** |
|  | 1. Provider Knowledge | 7 | *“I think that part of it is physicians. I think that not everyone is necessarily familiar with the recommendation that hepatitis B vaccine should be given to people with diabetes.”* ***–Participant 8***  *“Physicians are a barrier because like me, I'm not convinced that it's an actual need”* ***–Participant 6***  *“…the barriers that I came up with really were a lack of knowledge. And that's on the healthcare community.”* ***–Participant 9*** |
| **3b. Limited Electronic Medical Records (EMRs) Alerts** | 1. EMR/Follow Up | 11 | *“It's not something that, for example is, in Cerner, in my medical record, it's not one of the list of recommendations in Cerner when I have a patient with diabetes. They don't automatically say, "Hey, did this person get their?" But they do it for other things like their eye exams, their foot exams, things like that. It's not included in that type of recommendation in the recommendations pad, panel. That would mean that physicians that are unfamiliar with the recommendation, are not always, aren't aware because it's not in the recommendation panel. The second thing is that those that are aware, because it's not in the recommendation panel, can slip their mind a little easier because it's not there to be, to sort of highlight amongst the many other things we're dealing with that, "Hey, this person needs a hepatitis B vaccine."* ***–Participant 8***  *“But there is a special section where you can jump to that says vaccines... I don't even know the process. It doesn't always auto flag yes or no. So pretty much the practice is to just look. So, you look for every patient and see what do they due for and whatever they due for, you recommend.”* ***–*** ***Participant 9***  *“Well, because there are three doses that are often time things, people get busy, they forgot and I don't know. I think there's one thing that, the medical system is not as good as the dental system. We don't have the record, or the schedule where we send our reminder to our patients. So, oftentimes if we don't remind them, oftentimes they would just forget.”* ***– Participant 7***  *“You would think it would be easier given someone with diabetes is coming back fairly regularly, anyway. I think the challenge is, at least in our practices, you're so busy, so understaffed, and we don't have a system that automatically reminds us. And so, if I order the vaccine, it's not going to prompt automatically for them to schedule appointments. And the one of the staff people we're short on significantly in several clinics is the person who does the follow-up appointment schedule. So, they'll leave the office, then maybe two or three weeks later before someone can get them on the phone to schedule that appointment.”* ***– Participant 10*** |
| **3c. Competing Priorities** | - 1. Competing priorities | 6 | *“Again, I think it's just more attention towards other things related to their health. Even aside from vaccination in general, which I listed become more of a priority to the patient and myself, but the other aspects of their care, just they're plain diabetes management, their other comorbid health condition management, their prevent… their other non-vaccinated preventative health screenings related to colonoscopy and other cancer screenings and things like that. So, it just falls to the wayside.”* ***– Participant 1***  *“Yeah. At the point of care, we have as clinicians, as a care team, a lot of competing demands and we have to screen for their depression, and we have to monitor, get their blood pressure checked and if it's high, recheck it. We have to make sure we have all the laboratory data to be able to make the visit efficient. And then there are many parts of the care process that are recommended by the ADA that we will need to look at. But a subset of those are included in pay-for-value programs. So, there's an initial push at an office visit to make sure those things are attended to. And if there's time left over, then we might think about adding the other things like looking at their hepatitis B vaccination status and then updating it.”* ***–Participant 4*** |
|  | 1. Not a priority | 1 | *“But I think that might be a factor, is just we're not thinking about it as much, as a priority. Cause again, traditionally, training for most docs is that it's more just certain high risk-populations.”* ***–Participant 2*** |
| ***Subtheme: PCPs’ Perception of Patient-Level Barriers*** | | | |
| **`3d. Lack of Insurance Coverage** | 1. Insurance (General) | 4 | *“I mean… insurance companies won't cover it for most people, whether diabetics or not.”* ***–Participant 6***  *“I do think that insurance is likely a barrier and I will admit that I don't have a good sense of insurance coverage for hepatitis B vaccinations. And so that also is a reason that I don't always push for them because it's hard to determine that coverage. So certainly, that is a barrier that I've seen. And I don't know what category hepatitis B falls into in the past. I have been told that it is hard to get covered.”* ***–Participant 3*** |
|  | 1. Insurance (Diabetic) | 6 | *“I think insurance may be an issue. I think that individuals sometimes worry about whether their insurance is going to pay for the vaccination. Being within a health system where we have... Forget how we term the extra billing, but there's a facility fee I think is how we would term it. So, it's more expensive many times to get a vaccination within our health system than if they were to go to a pharmacy and get the vaccine there. So, I think the challenge with out-of-pocket cost may be an issue.”* ***– Participant 4***  *“Definitely income and insurance, for sure. That's always a barrier, just the economics of what we do, and just that barrier to care in general. Whenever there's an underserved, or uninsured, that's a huge barrier for those patients.”* ***–Participant 2*** |
| **3e. Patient Beliefs and Vaccine Hesitancy** | 1. Patient Vaccine Hesitancy | 7 | *“Then there are those who just have this really deep fear, they just don't want things, injection into their body, and then whatever fear that is either is a fear of the needles, or is it fear of something injecting into their body. Then there's also all kinds of crazy misinformation that goes around, people might say, if somebody has some bad experience with a vaccine, then tells everybody not to get vaccination. So, there's also that piece in it too.”* ***–Participant 7***  *“I think that it's not just hepatitis B that they're less likely to be vaccinated against, it's pretty much everything. And that just has to do with health literacy and vaccine hesitancy generally.”* ***–Participant 8***  *“I think there's some hesitance there on the patient side of things. Hepatitis B is not one of the ones that I frequently run into as what I would think about in my head as a difficult sell, but then again, there is hesitance in any of that population.”* ***–Participant 11*** |
|  | 1. Covid-19 Influence | 7 | *“Yes. I think that there are some people that have become more vaccine hesitant overall because of COVID vaccines.”* ***–Participant 3***  *“I think the other thing is COVID has really made this far more difficult to bring up, so. And I think it's not just the hepatitis B vaccines, it's come up with flu shot. Now we have a new pneumonia shot, early in the COVID there was a new Zoster shot. It's got everybody concerned about the safety of these, what's in these vaccines. A lot more people hesitant to want to get it.”* ***–Participant 10***  *“But I definitely think there's more of a barrier, and there's like a sense of vaccine fatigue right now from patients, because it's so prominent and present. And unfortunately, a lot of them are just closed off to that discussion because of unfortunately, sadly, because of everything with the COVID vaccine.”* ***–Participant 2***  *“In the healthcare community, we are facing a horrible, a horrible crisis and trust because of COVID. And we are struggling to convince people to take a COVID vaccine. And this distrust is spilling over into all of our recommendations. So, it's not entirely clear that recommending the hepatitis vaccine will help.“* ***–Participant 9*** |
| **3f. Lack of Patient Knowledge and Health Literacy** | 1. Knowledge (General) | 4 | *“But patients, unless they really understand, they don't see it as a disease that they've got to worry. But look at COVID disease, you can't get people to get COVID vaccine. So, hepatitis B doesn't scare people, really.”* ***–Participant 6*** |
|  | 1. Education/Knowledge | 10 | *“One, is the patient themselves not understanding why it's necessary. When you tell them it's bloodborne and you get it from needles or sexual contact, they're going to say, ‘Well, that doesn't apply to me.’”* ***– Participant 6***  *“I think the health literacy is a major challenge that we face. They might not even understand their diabetes and they might even less understanding what is hepatitis B or why is that important? With that group, we just need to spend more time to make sure they understand all the healthcare that they need and they deserve. I think they just need more education and more information.”* ***–Participant 7***  *“I don't know what the literature says about people understanding the importance of hepatitis B vaccination and individuals with diabetes, but maybe there's a knowledge gap. I can't say for sure, but I suspect that might be part of the issue.”* ***–Participant 4*** |
| **3g. Socio-demographics** | 1. Education/Knowledge | 10 | *“You know, on one hand I say yes, that maybe those with less education, they're more likely to be uninsured or underinsured and therefore not have access to preventative health and seeing a primary care provider. So that might be part of it”* ***–Participant 1***  *“Lower education folks are also less likely to have comprehensive insurance. They're more likely to live in a place with less access to healthcare.”* ***–Participant 9*** |
|  | 1. Age | 8 | *“I think the younger groups would have gotten the hepatitis B as part of their childhood immunization. I would think if they are going through the childhood immunization, they should have gotten it. Whereas, I think the older population, there would be some that haven't gotten it when they were children. I think we do tend to forget that there are some vaccination that we need to catch up the patient on.”* ***–Participant 7***  *“My own opinion would be that patients as they age are probably less likely to take the vaccine.”* ***–Participant 9***  *“I would say probably generally speaking, the older population is more receptive to vaccinations in general, and it's definitely more of the younger population that I do have more challenges with, overall.”* ***– Participant 2*** |
|  | 1. Other (General) | 9 | *“However, in conjunction with that, I do find the anti-vaccine movement in general, related to all vaccines, seems to come out of more the white privileged Christian faith background.”* ***–Participant 1***  *“I mean, all those social economic factors, they play a big role too.”* ***– Participant 5*** |
|  | 1. Other (Diabetic) | 8 | *“Race and gender... I would say gender not so much. I can't really see that just as a general rule I see that difference. Race maybe in lower SES status groups, which may tend to be certain races, just in general. That may apply. But I don't know if it's more the race, or it's more the socioeconomic status piece. My suspicion is it's more the SES piece, and not the race directly.”* ***– Participant 2***  *“I know that gender, especially in certain areas and certain clinics. I know age, especially with prejudice, assuming that they believe a certain way or they're going to act a certain way. Socioeconomic status is a big one because you can't have continuity care. It's really hard to get back in for those vaccinations and we know that. Obviously, any orientation identification, ethnicity questions, especially depending on your care and provider. There's certainly barriers there. And all of that will impact both A1c, clinical driven outcomes for diabetes and immunization, preventative care and all that stuff.”* ***–Participant 11*** |
| **Theme 4. Provider recommended strategies to increase hepatitis B vaccination among adults with diabetes** | | | |
| **4a. Providers Strategies** | 1. Provider Education | 4 | *“I think educating primary care physicians like myself about why should that be more of an important vaccine that we're recommending”* ***– Participant 1***  *“Probably, we need more physician education, physician and clinician information because I don’t think it’s widely understood as to why this is being recommended.”* ***–Participant 6***  *“I think just kind of focusing on the people that are the greatest touch point, which is primary care providers, and just really reinforcing and giving more education at the provider level first...”* ***–Participant 2*** |
|  | 1. Provider Evaluation | 3 | *“So, I know for our office, we have a lot of quality metrics that we keep good track of, and that is not one of them. And so, getting that added into our quality measures and into our checklist that we do with diabetic patients who certainly probably help.”* ***–Participant 3***  *“If you're being graded, there's more likely that you're going to have compliance from the providers. And it's going to be something that we're focusing on more. Not because we don't now, but to be honest, I mean primary care docs, we are so over-extended. And the thought of more on our plate, I think, is just so overwhelming. So, if it was something that was really a standard of care specifically, and was really reinforced, at maybe that level, it would probably help compliance by the providers, which would carry over to the patients.”* ***–Participant 2*** |
|  | 1. Provider Resources | 10 | *“I do think a thing that could be helpful is knowing that regardless of the practice setting in this country, we're often encouraged and we utilize diabetes educators in our office. So, I think if we were to increase uptake of Hep B recommendation and vaccination, having it within a diabetes education visit may be a more beneficial process where the nurse or the dietician doing the diabetes education have that as part of their education and also to be able to have a workflow if the patient was interested to set them up for Hep B immunization.”* ***–Participant 1***  *“I think that the conversation initiated by the MA or the nurse is highly effective. A great deal of vaccination in this country occurs because nurses promote it. So, I think it's very important. And we also know from adult learning that hearing information multiple times from multiple sources increases your probability of believing it…So if we train people on how to do the communication and repeat the communication, we should have better outcomes. And automatic flag is an easy way to take that off the plate of people who are busy.”* ***–Participant 9***  *“I think it would be really good if it is included in the guidelines, just like we recommend the pneumonia vaccine, we need to include that in their guideline as well. Then it will be continue to remind the patient and the physician that there is another vaccine that they should get. At some point I always, I may be too ambition wanting our electronic health record to do more reminding for us, so if there is a reminder that pops up or send us an alert, that this patient is needing to have X, Y, and Z, I think that would help us physician to do a better job in making sure all patients get the care they need. I think it could go beyond vaccination too, it could go into, time for their mammogram or time for the colorectal screening.”* ***– Participant 7*** |
| **4b. Patient Strategies** | 1. Patient Resources | 8 | *“If we could have some developed resources, would be good, it could be as simple as a flyer or a brochure. If we could develop some kind of a video or something, they can watch, they can explain, that would be great. We have care managers, or we're trying to incorporate care manager into our practice. If we could have more of them or they become more available, it would be good if we could refer a patient to an educator or a coordinator who can spend a little more time to explain these things, because if they really have issue with health literacy, it's not going to be a couple minutes, you need to take time to explain it. Either technology, or it could be as simple as a piece of paper, but then they may not read it or they may not understand, or know how to read it. Maybe a video might work better. But definitely a healthcare person of some type, either a nurse, or a care manager, or someone who are knowledgeable in this area would even be better, but that's becoming very resource intensive.”* ***–Participant 7***  *“If you put it on TV, on commercials and say, "This is something you should do, ask your doctor," that would be one thing. The little TV I watch, I see all these advertisements for complex drugs that they put out there like the average layperson has any clue whatsoever whether they should actually, be taking those things.”* ***–Participant 6***  *“I do think social media is a pretty good platform nowadays for transferring information. So that might be a good resource. However, also it's a hindrance, I think sometimes, because there's a lot of misinformation.”* ***–Participant 2***  *“What will work is economic incentive. And that's trivially easy. An insurance company that says, oh yeah, we're going to drop your rates if you do this… If you show us an up to date, vaccine record for all vaccines… If you show a gym membership, right, which is free, you show attendance records, or you show a healthy BMI, we drop it. If you do those kind of economic incentives, it's no longer a government mandate. It's no longer a health mandate. It's a business decision. And Americans vote with their pocketbook. Compliance will go through the roof.”* ***–Participant 9***  *“I mean, organizing community camps regarding, let's say preventative thing, vaccinations for pneumonia, for hepatitis B, for pap smear. I mean, all those kind of things. You can go to the community, organize camps, maybe every once in every three months or once every six months in different places. Involve the community organization.”* ***–Participant 5*** |
